# Abdominal Pain in a Patient With Giant Cell Arteritis

**DOI:** 10.7759/cureus.24149

**Published:** 2022-04-14

**Authors:** Tharani Sundararajan, Krati Chauhan

**Affiliations:** 1 Department of Internal Medicine, Southern Illinois University School of Medicine, Springfield, USA

**Keywords:** microperforation, glucocorticoids, acute abdomen, abdominal pain, giant cell arteritis

## Abstract

Giant cell arteritis (GCA) is a large vessel vasculitis seen in the elderly. It is primarily treated with corticosteroids, which are known to have a multitude of adverse effects, including predisposition to infection and intestinal diverticular perforation. We describe a unique case of a GCA patient with the subtle presentation of acute abdomen. A 71-year-old woman with GCA on corticosteroids presented with vague abdominal pain at a routine follow-up appointment. Diagnostic workup revealed perforated diverticulitis and urinary tract infection. She was admitted and managed conservatively. Clinicians may encounter similar scenarios to ours in which GCA patients will present with subtle symptoms of an acute abdomen. Corticosteroids mask symptoms in the setting of severe complications, especially in elderly patients. We recommend providers have a high index of suspicion for an acute condition, even when the clinical manifestations are subtle.

## Introduction

Giant cell arteritis (GCA) is a systemic vasculitis with a median age of onset of 75 years, primarily managed with high-dose corticosteroids. Infections are of significant concern in these patients as both the nature of the disease and its primary method of treatment weaken immunity [1]. Corticosteroids increase the risk and mortality from colonic diverticular perforation [2]. Kaya et al. previously described a GCA patient taking around 50 mg prednisone daily, a significantly higher dose than our patient who presented with abdominal symptoms, fever, and tachycardia at an emergency department [3].

To our knowledge, this is a unique case of a GCA patient taking 15 mg of prednisone daily who was diagnosed with a perforated colon at a routine outpatient follow-up appointment. She had normal vital signs with no signs or symptoms of peritonitis other than mild abdominal pain.

## Case presentation

A 71-year-old woman with an 8-year history of GCA presented to a routine rheumatology visit. She had previously been stable on methotrexate, 20 mg, and leucovorin, 5 mg weekly. Eight weeks ago, the patient was started on a prednisone taper starting at 60 mg daily for a GCA flare manifesting as headaches and elevated levels of C-reactive protein (CRP), 255 mg/dL, and erythrocyte sedimentation rate (ESR), 79 mm/hr. She was seen by ophthalmology and the eye examination was unremarkable. Her headaches resolved, and inflammatory markers trended down, with CRP down to 10 mg/L and ESR to 5 mm/hr.

At this visit, she reported new-onset abdominal pain for the last five days. It was non-radiating, crampy pain located in the bilateral lower quadrants and pelvic region. 4/10 in severity, relieved with acetaminophen, and not related to food or position. She denied a change in bowel habits, hematochezia, nausea, and vomiting. She was on 15 mg of prednisone at the time.

**Figure 1:**
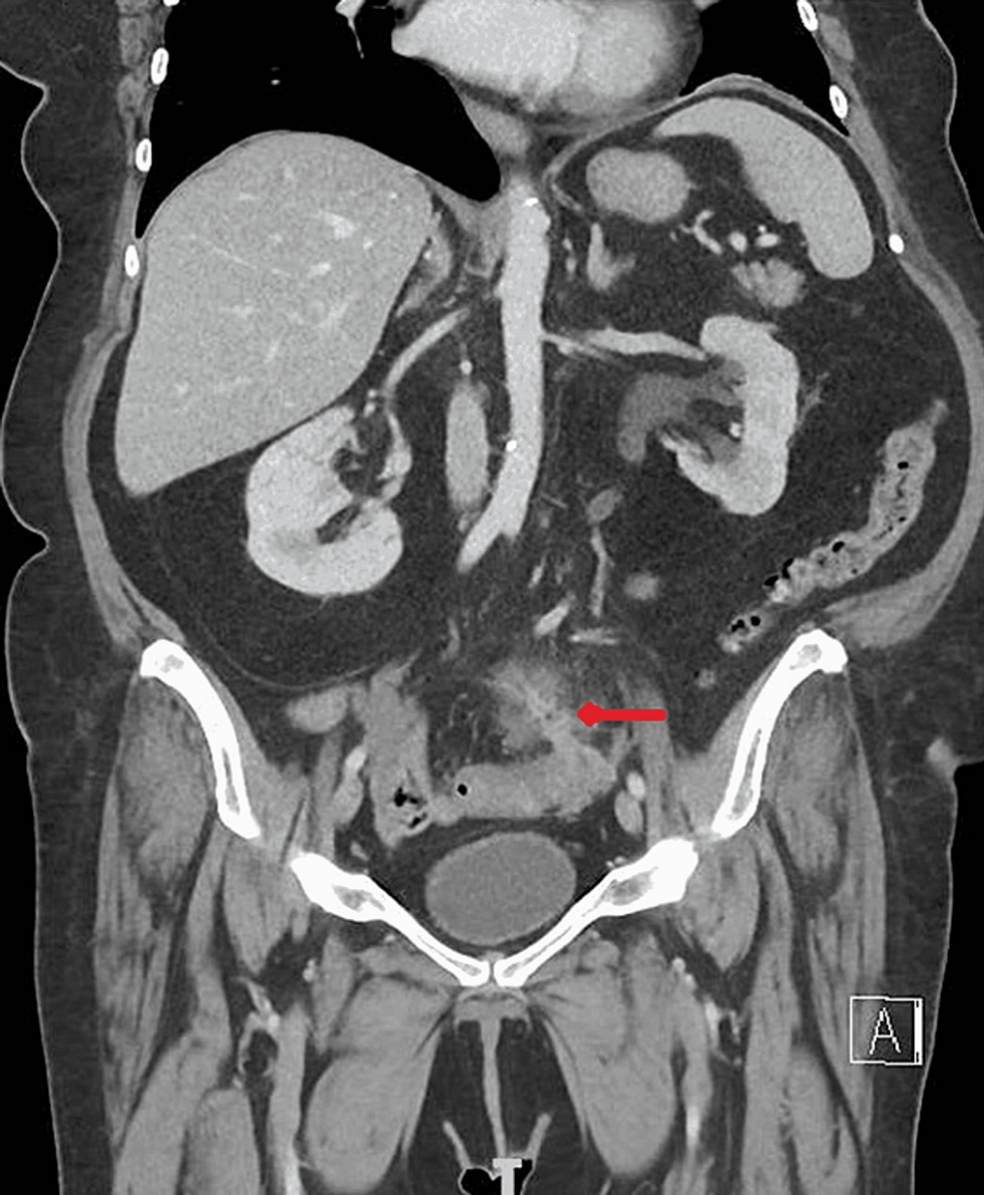
CT of the abdomen and pelvis showing focally perforated diverticulitis of the sigmoid colon (red arrow)

On physical examination, vitals were stable. Abdominal examination showed mild tenderness of bilateral lower quadrants with no guarding or rebound tenderness. The remainder of the systemic examination was unremarkable. Laboratory testing revealed a white blood cell count (WBC) of 13.5/mm^3^, hemoglobin of 11.7 g/dL, platelet count of 377/mm^3^, blood urea nitrogen (BUN) of 17 mg/dL, creatinine of 0.6 mg/dL, aspartate aminotransferase transferase (AST) of 11 units/L, alanine aminotransferase (ALT) of 16 units/L, CRP of 166 mg/L, and ESR of 81 mm/hr. Abdominal pain and elevated levels of CRP, ESR, and WBC were concerning, hence the patient was sent to the emergency department for further evaluation. CT of the abdomen and pelvis showed focally perforated diverticulitis of the sigmoid colon without drainable abscess (Figure 1). Urine analysis revealed nitrate positive urine with a WBC of 49/mm^3^ and many bacteria.

The patient was admitted for conservative management of perforated diverticulitis and urinary tract infection. She received empiric treatment with IV metronidazole and IV ciprofloxacin, which was changed to oral metronidazole as her condition improved. Urine culture grew klebsiella sensitive to levofloxacin, and IV ciprofloxacin was changed to oral levofloxacin. Prednisone was tapered and discontinued. Upon discharge, she completed two weeks of oral metronidazole and levofloxacin. Follow-up colonoscopy done eight weeks later showed two polyps in the ascending and descending colon. Biopsy reported tubular adenoma and hyperplastic polyp in ascending and descending colon, respectively. She has remained stable and continued methotrexate with leucovorin.

## Discussion

We have presented a 71-year-old female with GCA on high-dose corticosteroids (defined as > 7.5 mg of prednisone daily by the American College of Rheumatology) who developed perforated diverticulitis. Corticosteroids decrease the proliferation and migration of lymphocytes, impair the adhesion of neutrophils, inhibit cytokine secretion by macrophages, and induce thinning of the skin and mucous membrane, thus predisposing to infection [4,5]. Furthermore, corticosteroids disrupt the repair of the intestinal mucosa, inhibit local prostacyclin synthesis, and interrupt the immune response, thereby increasing bacterial colonization and the risk of diverticular rupture [3]. The frequency and mortality of colonic diverticular perforation are higher with corticosteroid use [2].

Diagnosis of infection is challenging in the elderly, and corticosteroids further confound the picture. Signs and symptoms of infection are masked because corticosteroids reduce pain and systemic manifestations of infection, such as fever [4]. A daily dose of > 15 mg of corticosteroids, which our patient was taking, double the infection risk, and one-third of these patients may be hospitalized [6]. Our case is noteworthy because it highlights the spectrum in which severe gastrointestinal complications may present in the setting of GCA being treated with corticosteroids. Thus, GCA patients must be managed with a high index of suspicion for complications from the corticosteroid use to prevent potentially devastating outcomes.

## Conclusions

GCA is a systemic vasculitis of the elderly treated with corticosteroids which may result in a range of complications. Clinicians must remain vigilant in identifying subtle signs and symptoms of infections in GCA patients. Elevation of ESR and CRP in GCA patients is not always due to GCA flare. It may be because of infection and patient management should be guided by history and examination findings.
